# Association between Body Composition, Physical Activity, Food Intake and Bone Status in German Children and Adolescents

**DOI:** 10.3390/ijerph17197294

**Published:** 2020-10-06

**Authors:** Juliane Heydenreich, Antje Schweter, Petra Lührmann

**Affiliations:** Institute of Health Sciences, University of Education Schwäbisch Gmünd, 73525 Schwäbisch Gmünd, Germany; julchenmade@gmail.com (A.S.); petra.luehrmann@ph-gmuend.de (P.L.)

**Keywords:** bone health, bone healthy eating index, physical activity level, children, adolescents, calcaneal stiffness index

## Abstract

Achieving a high bone mass during childhood and adolescence is important for the prevention of osteoporosis in later life. Herein, the purpose was to assess the relationship of various lifestyle factors and bone outcomes in school children. In 248 girls (13.4 ± 1.9 years, BMI: 20.2 ± 4.8 kg m^−2^) and 231 boys (13.6 ± 1.7 years, BMI: 19.3 ± 3.3 kg m^−2^), bone mass (stiffness index, SI; calcaneal quantitative ultrasonometry), body composition (bioelectrical impedance analysis), food intake (food frequency questionnaire), and physical activity level (PAL; standardized questionnaire) were assessed. The individual food intake of eight food groups was related to the German recommendations (Bone Healthy Eating Index, BoneHEI; 0–100 points). Relationships between SI and lifestyle factors (Spearman´s rank correlation) and the influence of the factors on the variance of SI (multiple linear regression) were tested (α = 0.05). SI correlated with age, BMI, absolute fat-free mass, relative fat mass, PAL, and puberty category score in both girls and boys (*r* = 0.18–0.56, *p* < 0.01), but not with BoneHEI (*p* > 0.05). Age, absolute fat-free mass, sex, and PAL explained 35% of the variance of SI (*p* < 0.0001): SI = −0.60 + 2.97∙age (years) + 0.65∙fat-free mass (kg) + 6.21∙sex (0 = male, 1 = female) + 17.55∙PAL. Besides age and sex, PAL and fat-free mass are important factors relating to bone health. School children should perform regular physical activity to improve their bone status.

## 1. Introduction

Osteoporotic fractures are an important cause of morbidity and mortality [1] and represent a high economic burden to society [2] as well as a reduced quality of life. The main cause of osteoporotic fractures is a reduced bone mass, which results from an age-related bone loss and/or failure to achieve optimal peak bone mass in the growing years [3]. Bone mass increases substantially during the first two decades of life and reaches a plateau (referred to as *peak bone mass*) in the late-teen or young-adult years [4]. Achieving a high peak bone mass in early life predicts a relatively higher bone mass, and hence greater fracture protection, late in life [5]. Up to 60% of the risk of osteoporosis can be explained by the amount of bone mineral acquired by early adulthood [6]. Besides genetic factors, lifestyle influences 20–40% of adult peak bone mass [7]. Accordingly, an important strategy to reduce the risk of osteoporosis or low bone mass in later life is the optimization of these factors known to influence peak bone mass and strength.

Important variables influencing bone health include the anthropometric status and body composition. Body weight (which is itself a compound of environmental and hereditary factors) is the largest single determinant of the variability in adult bone mass [5]. For example, a very low body mass index (BMI) is associated with a higher risk of osteoporosis [8]. On the other hand, it has been reported that high levels of muscle mass are beneficial for bone mass due to the mechanical and biochemical coupling between muscle and bone [9].

In addition, regular physical activity is one important factor for improving bone health and bone mineral density [10]. Bones adapt to the load applied to them, so increased mechanical loading leads to an increase in bone density whereas the removal of customary loads is followed by bone loss [11]. Therefore, intense and repeated activities with rapid changes of direction, accelerations, and/or jumps are key determinants for bone mineral density during growth [12].

Among other factors, calcium and vitamin D deficiencies can lead to a decrease in bone mineral density and a predisposition to osteoporosis [5]. Vitamin D insufficiency is usually the result of inadequate sunlight exposure and/or low dietary intake [5]. Therefore, in some studies, positive associations between sunlight exposure and bone status are reported in adolescents [13,14]. Finally, early-life breastfeeding is associated with a beneficial increase in bone mass and a reduction in fracture risk during adolescence [15]. 

There are currently only a few studies assessing the influence of multiple parameters on bone health in children and adolescents available, with most of them focused on a limited number of lifestyle factors [13,14,15,16,17]. Therefore, the purpose of this study was to examine the relationship between several lifestyle factors and bone parameters in boys and girls.

## 2. Materials & Methods

### 2.1. Study Design and Participants

The present study is a cross-sectional study with a non-representative sample of school children. Recruitment of participants was performed on three levels of selection: (1) recruitment of schools, (2) recruitment of teachers and classes, and (3) recruitment of school children. At a conference of 18 headmasters from secondary schools in Schwäbisch Gmünd, Germany, and surroundings (autumn 2009), the project leader (PL) presented information about the present study. Two headmasters immediately gave their permission, whereas four headmasters agreed after personal communication at a later date for participation of their secondary school in the study. All recruited schools received further information about the study. Teachers of the participating schools were informed by announcements and personal communication in order to recruit classes for data assessment. Each student from the recruited classes (*n* = 954) received a letter with detailed information regarding the purpose and methods of the study and written informed consent, which had to be signed by the parents. Only school children who had provided written informed consent were included in the study. In total, 486 volunteers participated in the study. The response rate was thus 50.9%.

Measurements took place between June 2010 and July 2011 on one testing day in a separate room provided by the respective school. Before testing, volunteers were questioned regarding how they feel. Only school children who stated that they feel good and are not ill were included in the study. In addition, volunteers who were amputated or have a bridge were excluded, since analysis of body composition could not be conducted. The volunteers were tested in the following order: (1) anthropometry and body composition and (2) bone status (see details below). In addition, volunteers filled out a standardized questionnaire to assess their lifestyle habits and dietary intake (see details below). Furthermore, before investigations, parents of the volunteers had to fill out a standardized questionnaire. The study was approved by the University’s Ethics Committee, and the study was carried out according to the recommendations of the Helsinki Declaration (2008).

### 2.2. Anthropometric Data and Body Composition

Volunteers were in light clothing and without shoes when body mass was measured to the nearest 0.1 kg using a calibrated beam scale (Seca 877, Seca, Hamburg, Germany). Afterwards, 0.5–1.0 kg was subtracted from the measured body mass, depending on the estimated weight of the subject wearing clothes. Height was assessed to the nearest 0.5 cm using measuring tape, with volunteers standing and looking straight on without wearing shoes. BMI was derived from weight divided by height in meters-squared and classified according to age- and sex-specific percentile curves for German children and adolescents [18]. Children/adolescents with a BMI < 3rd percentile were considered as strongly underweight, < 10^th^ percentile as underweight, between 10th and 90th percentile as normal weight, > 90th percentile as overweight, and > 97th percentile as obese [18].

Body composition was assessed taking whole body bioelectrical impedance measurements (resistance, *R*, and reactance; *Xc*, at 50 kHz and 800 µA) using an impedance analyzer (BIA 2000 - S, Data Input GmbH, Pöcking, Germany). Measurements were performed in the morning with volunteers in light clothing, bladder-voided, and all metal artefacts removed. School children were in supine position with legs and arms abducted 45° from the body during the measurement. After the skin was cleaned with an alcohol wipe, two pairs of detector and injector electrodes were placed on the right hand and foot [19]. During the measurement volunteers were requested not to move. Absolute fat-free mass was then calculated using the equation of Plachta-Danielzik et al. [20]. According to the manufacturer, the precision data are 0.5% for *R* and 2.0% for *Xc*. Almost perfect correlations (*r* = 0.81–1.00) between fat mass and fat-free mass estimated by bioelectrical impedance analysis and reference methods were obtained in male and female children and adolescents in a recently published systematic review [21].

### 2.3. Bone Status

Bone status was assessed using quantitative ultrasound (QUS) measurements with the Lunar Achilles InSight Ultrasonometer (GE Healthcare, Milwaukee, WI, USA) and analyzed using the accompanying software. QUS measurements use ultrasound waves to measure broadband ultrasound attenuation (BUA; dB MHz^−1^) and the speed of sound (SOS; m s^−1^). According to the manufacturer, the stiffness index (SI) is then calculated using the following equation:

SI = (0.67 ∙ BUA) + (0.28 ∙ SOS) − 420.(1)

Z-score values of BUA and SOS were calculated using the reference sample of a healthy, pediatric German sample stratified by age and sex [22] by using the following equation:

*Z*-score = (measured values − matched mean values) / matched SD. (2)

A Z-score of −2.0 standard deviation or lower was defined as “below the expected range for age” and a Z-score above −2.0 as “within the expected range for age” [23].

All measurements were performed and analyzed by trained researchers and standardized according to the manufacturer´s recommendations. School children were comfortably sitting on a stable chair directly in front of the Achilles device. The left leg was placed in the device, so that foot, calf, and thigh aligned with the calf support and the positioner. They were requested not to move during the measurement. Calibration of the Achilles device was performed on a weekly basis using a calibration phantom. The Achilles densitometer has been shown to provide good precision for SI (<2%) in children and adolescents [24,25]. Significant correlations between the SI measured by quantitative ultrasound and outcomes measured by dual energy X-ray absorptiometry have been reported in children and adolescents (*r* = 0.69; *p* < 0.001) [24].

### 2.4. Lifestyle Questionnaire

Volunteers were requested to fill out a standardized questionnaire, where demographical data, physical activity, and staying outside during summer/winter and week days/week-end (h d^−1^) were questioned (Supplementary Materials).

In the questionnaire, volunteers recorded their average time spent engaging in low-, medium-, and high-intensity physical activities for one week, for example, for housework and gardening, walking, cycling, sporting exercise, and others [26]. In addition, the average daily time spent for sleeping and media use was documented. The time spent for preparation and participation of school courses was estimated to be 30 hours per week. Volunteers where the sum of the duration reported for all activities was higher than 24 h per day were excluded from analysis for physical activity level (PAL). Resting metabolic rate (RMR) of the volunteers was calculated using the equation of Müller et al. [27]:

RMR (MJ d^−1^) = 0.02606 ∙ weight (kg) + 0.04129 ∙ height (cm) + 0.311 ∙ sex (female = 0, male = 1) − 0.08369 ∙ age (years) − 0.808. (3)

The total energy expenditure was then estimated by multiplying specific factors for each activity with the estimated RMR [28]. Finally, PAL was calculated by dividing total energy expenditure with estimated RMR.

Volunteers estimated their physical development using a validated questionnaire consisting of five items (growth in height, body hair, skin changes, facial hair, and voice changes for boys; breast development and menses for girls) [29]. Each item had to be evaluated with an adapted 4-point-scale (1 = not yet started, 2 = barely started, 3 = definitely started, 4 = seems completed) [30]. With the three salient sexual maturation characteristics of the pubertal development scale (menarche, breast and body hair growth in girls, and deepening-voice, body hair, and facial hair growth in boys), puberty category scores were computed in order to categorize the children into one of the five pubertal development stages (pre-, early-, mid-pubescent, advanced, and post-pubescent) designed to be similar to Tanner staging categories [31]. 

In addition, parents of the school children had to fill out a standardized questionnaire, where they had to answer questions about the vitamin D supplementation in the first year of life, the regular drug intake, and the history of fractures of their child. 

Reproducibility of the physical activity questionnaire and food frequency questionnaire was previously checked in 102 boys and 90 girls [32]. We found significant positive correlations for reported physical (in-) activities (*r* = 0.38−0.74; all *p* < 0.01) and food groups (*r* = 0.46−0.72; all *p* < 0.01) between the first and second assessment. Therefore, with regard to food intake and physical activity, the questionnaire can be considered reproducible.

### 2.5. Food Intake

Food intake was assessed using an adapted version of the standardized food frequency questionnaire “What do you eat?” [33] (Supplementary Materials). The chosen 13 food items reflected the main sources of calcium, vitamin D, protein, phosphate, and energy observed in a study of 10–17 years old boys and girls [34]. Volunteers had to estimate the average frequency and the usual amount of these food items ingested within the last weeks. The response options for frequencies were identical for all food items: never, once per month, 2–3 times per month, 1–2 times per week, 3–4 times per week, 5–6 times per week, once per day, 2–3 times per day, 4–5 times per day, more than 5 times per day. The amount of food ingested was estimated using standard household measured of the food items, e.g., a handful, 1 slice, 1 glass (in total, 5 response options) and converted into g amounts. Experts recommend the use of a food-based instead of a nutrient-based index to consider the diet as a whole, as well as taking into account current recommendations, and to consider diet variety [35]. Therefore, a Bone Healthy Eating Index (BoneHEI) was calculated based on the Healthy Nutrition Score for Kids and Youth (HuSKY) [36], taking into account the actual guidelines for an optimized mixed diet for children and adolescents [37,38]. For this, the questioned food items were summarized into eight different food groups (fruits and vegetables, fish, bread, milk and dairy products, meat and sausages, tolerated food, soft drinks, and caffeinated beverages; Table 1) [32]. The information regarding the food frequencies was recoded into times of servings per day. The daily intake (g d^−1^) of these food items was calculated as the product of amounts of the given portion sizes of the food items (g) and the servings per day. The individual intake was related to the recommended daily intake of these food groups [36,37,38]. For most of the food items (except meat and sausages, tolerated food, soft drinks, and caffeinated beverages) an intake below the recommendation was assessed proportionally (e.g., an intake of 70% of the daily recommended intake resulted in 70 points). For fruits and vegetables and fish, the participant reached 100 points when the recommended intake was reached or exceeded. For bread, 100 points were given if the intake reached or exceeded the recommendation up to the double recommended amount. When the ingested amount was higher than twice as recommended, points were proportionally subtracted from 100, because of the potential high energy contribution. For milk and dairy products points were proportionally subtracted from 100 when a participant exceeded the recommended intake. If volunteers ingested below or equal amounts of recommendations for meat and sausages, tolerated food, soft drinks, and caffeinated beverages 100 points were given. If the intake was higher points were proportionally subtracted from 100, since intakes higher than recommended are considered unfavorable. The BoneHEI was then calculated as the average of the points reached for each category (range 0–100 points). A higher BoneHEI reflects a healthier diet for the bone.

### 2.6. Statistics

Statistical analyses were performed with SPSS statistics version 26 for MS-Windows (IBM Corp., Chicago, IL, USA). Mean values and standard deviations (SD) were calculated and data was checked for normality using the Shapiro-Wilk-test. Sex differences were tested by Mann–Whitney U tests. Differences of SI between BMI categories were assessed using Kruskal–Wallis-tests. Results are considered statistically significant when *p*-values are <0.05. The relationship between bone parameters and several variables were first investigated using Spearman´s rank correlation analysis. The correlation coefficients (*r*) were classified according to Cohen [39]. An *r* between 0.10 and 0.29 was considered as small, between 0.30 and 0.49 as moderate, and between 0.50 and 1.0 as a strong association. Lastly, a multiple linear regression was performed, where SI was entered as dependent variable and significant factors observed during Spearman´s rank correlation as independent variables.

## 3. Results

### 3.1. Participants

Seven out of 486 volunteers were excluded from final analysis (two boys with missing questionnaire, one growth-restricted girl, two girls, and one boy with implausible nutrition data, one girl with missing bone parameters). In total, data of 231 boys and 248 girls were included in the analysis. In Table 2, anthropometric data and body composition as well as further characteristics for all volunteers are displayed. Boys had significantly higher values for height, absolute and relative RMR, and stayed longer outside than girls. The absolute and relative fat mass and the puberty category score was higher in girls compared to boys.

### 3.2. Food Intake

In Table 3, the intake of food components of the BoneHEI, the national average intake [40], and adapted dietary recommendations [37,38] are presented. Girls ingested significantly more fruits and vegetables than boys, whereas boys consumed more bread, fish, meat and sausages, and soft drinks than girls. Boys and girls ate fewer fruits and vegetables, bread, and milk and dairy products than recommended. In girls, the intake of fish was below, and the consumption of tolerated foods was higher than the recommended intake of these food groups. Boys ingested more fish, meat and sausages, and soft drinks than recommended. The total BoneHEI score was significantly higher in girls.

### 3.3. Bone Status and Influencing Factors

**Figure 1:**
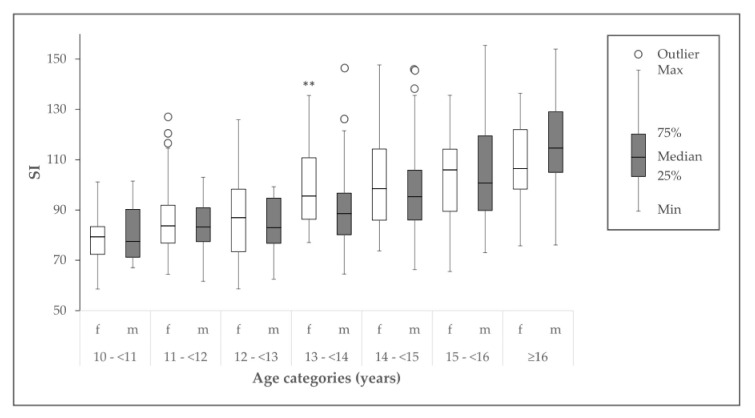
Calcaneal stiffness index (SI) of different age categories of boys (m) and girls (f). ** Significantly different from boys (*p* < 0.01).

In Table 4, the observed bone status parameters are displayed. There was no significant sex difference for SOS, BUA, and SI. In boys, the SI was not significantly different between BMI groups (*p* = 0.42), whereas obese girls had significantly higher values of SI compared to strongly underweight girls (102.2 ± 19.2 vs. 76.6 ± 17.6; *p* < 0.05). In Figure 1, the SI of girls and boys across different age groups are displayed. Girls had a significantly higher SI in the age group 13–<14 years compared to boys, whereas the SI was not significantly different in all other age groups.

In both boys and girls, no significant difference of SI was detected between volunteers who experienced at least one fracture during their life and those who did not, taking regularly medication and not, those who were breastfed during infancy and not, and those who took vitamin D supplements in their first year of life and those who did not (all *p* > 0.05).

In Table 5, the correlation coefficients of bone parameters with different factors are displayed. The SI strongly correlated with age, absolute fat-free mass, and puberty category score in both girls and boys (*r* = 0.52−0.56). The correlation between SI and BMI was “moderate” (*r* = 0.32−0.44), and between SI and relative fat mass and PAL was “small” (*r* = 0.18−0.27). In boys, a small but significant association between SI and the duration of breastfeeding was found, whereas in girls, a moderate relation between absolute fat mass and SI was obtained. In both sexes, no significant correlation between BoneHEI and SI was found.

In a first stepwise multiple regression model, the significant factors associated with SI identified during Spearman´s rank correlation were entered as independent variables: sex, age, BMI, absolute fat-free mass and fat mass, relative fat mass, PAL, puberty category score, staying outside, and duration of breastfeeding during infancy. SI was entered as dependent variable. BMI, absolute and relative fat mass, puberty category score, and duration of breastfeeding during infancy did not contribute significantly to the multiple regression model and were therefore excluded from analysis. Age, absolute fat-free mass, sex, and PAL explained 35% of the variance of SI (Table 6).

## 4. Discussion

The aim of this study were to examine the relationship between body composition, physical activity, food intake, other lifestyle factors, and bone parameters in male and female school children. We found that age, absolute fat-free mass, sex, and PAL were significant predictors of SI. 

Most of the volunteers of the present study were healthy, normal weight, breastfed and vitamin D supplemented during infancy, and stayed outside regularly. Their diet, in general, is comparable to the diet reported in the general population [40]. In addition, the BoneHEI can be regarded as being in the medium range when we compare the values with the weighted mean value of the original HuSKY-index in children and adolescents [36]. Therefore, the diet of the present study volunteers can be regarded as adequate. Hence, we assume that the lifestyle is rather favorable for bone health. This could also be the reason for the relatively good bone parameters of our study volunteers (girls and boys: SI = 94). In 177 school children (56 girls and 121 boys; age range 11–18 years) from a German college of physical education mean values of the total sample for BUA and SOS of 69.7 dB MHz^−1^ and 1581.1 m s^−1^ were reported, respectively [41]. By applying the equation for the calculation of SI used in the present study, a mean value for SI of ~69 was estimated. In the present study, we found much higher values for SI for both sexes. In addition, when we compared the values of SI of our volunteers with those from an age- and sex-matched German reference sample (1623 girls and 1676 boys; age range 6–18 years) [22], girls and boys of the present study had 47% and 60% higher values of SI, respectively. As concluded in a systematic review, the validity of quantitative ultrasound measurements strongly depends on the type of scanner used (aimed at assessing cortical or trabecular bone) produced by different manufacturers, and also which comparators were used (e.g., dual-energy X-ray absorptiometry vs. biochemical parameters) [42]. Therefore, one possible explanation for the huge difference of SI values is that, in both mentioned studies, a different qualitative ultrasound measurement device was used in comparison to our study. They also excluded girls using oral contraceptives, whereas we included them. Another explanation is that volunteers of the present study might have a more favorable lifestyle for bone health compared to study participants of the mentioned studies [22,41]. Unfortunately, for these studies, no data for both PAL and dietary intake are available.

In children it is particularly important to use Z-scores, not T-scores, for the interpretation of bone parameters [23]. In five girls (2.0%) and one boy (0.4%) of the present study, we found SOS Z-score values ≤ –2.0 standard deviation, indicating a value “below the expected range for age”. These individuals are at high risk for suffering from osteopenia or osteoporosis in later life. Up to know, there have been no data available for the prevalence of “low bone quality” in healthy German children and adolescents. Overall, the prevalence seems to be low, even when compared to data from other countries. For example, among 1590 Chinese students (11–17 years), 7.3% of the girls and 4.4% of the boys living in an urban environment were classified as having a “low bone quality” [43], whereas in 196 Korean boys and girls (12–15 years), 3.1% were classified as having a “low bone quality” [16]. In 250 Egyptian children and adolescents, 14.8% had abnormal bone health, most of whom were girls (67.6%) [13]. In this study, the authors reported in their participants a high prevalence of underweight, which is a risk factor for osteoporosis [8]. In addition, “poor bone health” was defined as a Z-score ≤ −1.5 standard deviation. However, besides different classifications of “low bone quality,” different assessment methods for bone quality (e.g., dual-energy X-ray absorptiometry vs. quantitative ultrasound measurements) might also explain the discrepancy in prevalence numbers for “poor bone health”. Finally, as mentioned above, the lifestyle of volunteers of the present study might positively influence bone health and is thus leading to low prevalence rates of “poor bone health” in comparison to the mentioned studies. We found that BMI and body composition parameters were related to bone parameters. However, only fat-free mass was a significant positive predictor of SI, as shown in the multiple regression analyses. The basic mechanisms underlying this relation remains unclear. One explanation might be that higher levels of fat-free mass are associated with a greater amount of physical activity and therefore, increase bone loading and thus bone density [11]. Another explanation might be that a larger body mass *per se* imposes a greater mechanical loading on the bone, and bone mass increases to accommodate the greater load [44]. We also found a positive association with PAL and bone parameters. These findings support again the theory that increased bone loading due to higher amounts and intensity of physical activity improve bone metabolism and are leading to a higher bone density [11]. In very active German children and adolescents, it was also shown that higher values of bone parameters were found in comparison to a reference sample [41]. These results again show the importance of regular physical activity for optimal bone growth.

As expected, we found a strong correlation between age and bone parameters. Age was also a significant predictor of SI in the multiple regression analysis. Since age and puberty are strongly related, it is not surprising that we also found a strong correlation between puberty category score and all bone parameters in both girls and boys. However, the puberty category score was not a significant predictor for SI in multiple regression analyses. In 250 Egyptian children and adolescents (8–18 years) the factor age was also a significant predictor on bone health [13]. Besides age, the authors also found that sex, demographic parameters, adiposity, physical activity, vitamin D and calcium intake, sun exposure, and several blood parameters were related to bone status and explained 88.4% of the variance of bone parameters among school children with normal bone status.

In Korean adolescents, those participants from the highest tertile of a “milk and cereal” dietary pattern score had a significantly reduced likelihood of having low bone mineral density compared with those in the lowest tertile [16]. In a recent systematic review, the relation between dietary pattern and bone mineral density in different age groups was assessed [45]. They found that, in children and adolescents, a comparison of the highest to lowest category of a “prudent/healthy” dietary pattern resulted in an inverse association with low bone mineral density. In contrast to these studies, we found no association between BoneHEI and SI.

The duration of staying outside was positively related to BUA in boys, but not in girls. In a study by Alghadir et al., sun exposure was associated with bone outcomes in male and female children and adolescents [13]. However, we assessed the average time spent outside and were not able to estimate the time the volunteers were exposed to the sun. 

We also found that only in boys the duration of breastfeeding was positively related to BUA and SI. However, the factor was not significant in multiple regression analyses. In the literature, it is reported that children at the age of six who were never breastfed had a lower bone mineral density compared to those who were breastfed [17]. However, the authors also found that, among all breastfed children, those who were breastfed non-exclusively in the first four months had higher bone mineral density compared with children breastfed exclusively for at least four months. The duration of breastfeeding was not associated with bone outcomes. When they adjusted the data for vitamin D supplementation in the first year of life, the same results were obtained. In the present study, we also found no statistical difference of SI between those volunteers who took vitamin D supplements in their first year of life and those who did not. Therefore, the influence of breastfeeding on bone outcomes in later life is not yet resolved definitively.

The results of the multiple regression analysis indicated that in healthy children and adolescents with a favorable lifestyle the factor age is the primary predictor of SI. The factor sex was also a significant predictor of SI, reflecting the fact that SI development is different between girls and boys, with the SI of girls increasing earlier in life compared to boys. Finally, fat-free mass and PAL were also important factors, highlighting the importance of regular physical activity for bone development.

### Strengths and Limitations

Strengths of the present study include that we were able to assess and analyze multiple parameters in a large number of subjects (*n* = 479). This is one of the few studies where multiple lifestyle factors, such as physical activity habits, dietary factors, habitual staying outside, use of medication, history of fractures, and breastfeeding during infancy were related to bone parameters in school children. 

However, we have to address some limitations. At first, the assessment of bone parameters must be discussed. We used quantitative ultrasound measurements to assess bone parameters in the study population. At present, dual-energy X-ray absorptiometry is the reference standard and the most widely used method to assess bone mineral content and bone mineral density. However, Torres-Costoso et al. reported that quantitative ultrasound measurements (using the same device as in the present study) could represent an acceptable alternative method to assess bone health in active adolescent males [46]. In addition, it is considered to be a safe, easy-to-use, radiation-free, cost effective, and portable method to assess bone parameters [47]. 

We also have to mention the assessment of physical activity as another limitation in the present study. Girls and boys estimated their daily physical activity based on a structured questionnaire [26]. Self-reported measures of physical activity show a greater level of variability compared to objective measures [48]. Furthermore, for the calculation of PAL, we used estimated RMR values obtained by the equation of Müller et al. [27]. RMR equation formulas often under- or overestimate true energy costs at rest at an individual level [49]. However, the equation used in this study was specifically developed for German children and adolescents aged 5–17 years [27]. 

Finally, we do have to address the limitation of the assessment of food intake. We chose an adapted version of a standardized food frequency questionnaire [33] for the assessment of 13 food items relating to bone health in boys and girls. By focusing on these food items, it might be that other food items also relating to bone health were missed. On the other side, we were able to assess and analyze bone-relating food groups in a large number of subjects.

## 5. Conclusions

In the present study age, absolute fat-free mass, sex, and PAL were significantly related to bone outcomes. Multiple regression analyses indicated that these factors were also significant predictors of SI. Physical activity and concomitant higher levels of fat-free mass are very important for bone status in school children. Therefore, we recommend the performance of regular physical activity to improve bone health and to reduce the risk of osteoporosis in later life.

## Figures and Tables

**Table 1:** Components and assessment of the Bone Healthy Eating Index (BoneHEI).

Food Group	The Following Items Are Included in the BoneHEI: “How Often Do You Consume…”	Allocation of Points
Fruits and vegetables	Fresh/boiled/preserved/frozen fruits; cooked vegetables (prepared from fresh, frozen, preserved vegetables)/salad/raw vegetables	I/R ≤ 1: proportional points up to 100 I/R > 1: 100 points
Fish	Fish	
Bread	Bread; bread roll	I/R ≤ 1: proportional points up to 100 I/R > 1 and ≤ 2: 100 points I/R > 2: points proportionally subtracted from 100
Milk and dairy products	Milk/cocoa/yoghurt/curd/buttermilk; cheese; cream cheese	I/R ≤ 1: proportional points up to 100 I/R > 1 and ≤ 2: points proportionally subtracted from 100 I/R > 2: 0 points
Meat and sausages	Meat; sausages/ham	I/R ≤ 1: 100 points I/R > 1 and ≤ 2: points proportionally subtracted from 100 I/R > 2: 0 points
Tolerated food	Sweets/chocolate/chocolate bar/cake/pastry/cookies/drops/fruit gums; snacks/chips/salt sticks/cracker	
Soft drinks	Coke/lemonade/soft drinks/energy drinks/iced tea	
Caffeinated beverages	Coffee/black tea/green tea	

I/R = intake (I) / age- and sex-specific recommendation (R) ratio for the food group. Modified from Kleiser et al. (2009) [36] and Schweter (2015) [32].

**Table 2:** Characteristics of the study volunteers. Data are presented as Mean ± SD and percentage.

Characteristics	Girls (n = 248)	Boys (n = 231)
Age (years)	13.4 ± 1.9	13.6 ± 1.7
Body mass (kg)	51.3 ± 14.8	51.0 ± 13.7
Height (cm)	158 ± 10	161 ± 13 *
BMI ^a^ kg m^−2^ Strongly underweight (%) Underweight (%) Normal weight (%) Overweight (%) Obese (%)	20.2 ± 4.8 2.4 6.0 73.4 9.7 8.5	19.3 ± 3.3 3.5 7.4 77.5 8.2 3.5
Fat-free mass (kg)	37.6 ± 7.2	41.6 ± 10.4 ***
Fat mass (kg)	13.5 ± 7.9	9.6 ± 5.6 ***
Fat mass (%)	24.9 ± 8.1	18.0 ± 7.3 ***
PAL ^b^	1.4 ± 0.1	1.5 ± 0.2 ***
RMR ^c^ (kJ d^−1^) (kcal kg^−1^ d^−1^)	5952 ± 618 29.3 ± 5.1	6345 ± 746 *** 31.2 ± 5.3 ***
Puberty category score ^d^ Prepubescent (%) Early (%) Midpubescent (%) Advanced (%) Postpubescent (%)	8.4 ± 2.9 3.5 10.5 18.6 57.0 10.5	7.0 ± 2.5 *** 10.2 20.9 36.2 31.1 1.7
History of fractures (%)	22.6	26.6
Use of medication (%)	6.5	7.9
Staying outside (h d^−1^)	3.6 ± 2.1	5.0 ± 5.6 **
Breastfeeding ^e^ Prevalence (%) Duration (months)	87.4 7.0 ± 4.2	83.8 7.8 ± 5.1
Vitamin D supplementation ^f^ (%)	72.7	73.5

BMI = body mass index, PAL = physical activity level, RMR = resting metabolic rate. ^a^ Classification according to Kromeyer-Hauschild et al. (2001) [18]. ^b^ Data presented for 220 girls and 174 boys. ^c^ RMR predicted by the equation of Müller et al. [27]. ^d^ Data presented for 172 girls and 177 boys. Pubertal development stages were categorized according to Crockett (1886) [31]. ^e^ Breastfeeding during infancy. ^f^ Vitamin D supplementation during first year of life (“often” + “frequently”). * Significantly different from girls (*p* < 0.05). ** Significantly different from girls (*p* < 0.01). *** Significantly different from girls (*p* < 0.0001).

**Table 3:** Components of the Bone Healthy Eating Index (BoneHEI), their intake of volunteers of the present study (*n* = 248 girls, *n* = 231 boys), the national average intake [40], and adapted dietary recommendations [37,38]. Data are presented as Mean ± SD.

Food Group	Intake of Children	National Average Intake (14–18 years)	Recommended Intake (15–18 years)	BoneHEI Score
Fruits and vegetables (g d^−1^)				
Girls Boys	471 ± 661 387 ± 516 **	323263	600 g d^−1^ 700 g d^−1^	57 ± 33 45 ± 34 ***
Fish (g d^−1^)				
Girls Boys	7 ± 14 17 ± 40 ***	5 6	100 g wk^−1^ (14 g d^−1^)	31 ± 39 47 ± 43 ***
Bread (g d^−1^)				
Girls Boys	115 ± 136 170 ± 188 ***	142 182	280 g d^−1^ 350 g d^−1^	37 ± 31 43 ± 32 *
Milk and dairy products (g d^−1^)				
Girls Boys	337 ± 377 381 ± 418	240 330	450 g d^−1^ 500 g d^-1^	37 ± 29 42 ± 32
Meat and sausages (g d^−1^)				
Girls Boys	75 ± 103 166 ± 198 ***	57 104	75 g d^−1^ 85 g d^−1^	76 ± 39 52 ± 45 ***
Tolerated food (g d^−1^)				
Girls Boys	77 ± 175 118 ± 271	69 81	1 serving d^−1^ (48 g)	75 ± 39 70 ± 43
Soft drinks (mL d^−1^)				
Girls Boys	347 ± 694 563 ± 858 ***	260 505	200 mL d^−1 a^	76 ± 42 60 ± 47 ***
Caffeinated beverages (mL d^−1^)				
Girls Boys	106 ± 322 103 ± 333	118 116	150 mL d^−1 a^	90 ± 30 92 ± 27
Total BoneHEI				
Girls Boys				60 ± 13 56 ± 16 *

^a^ No recommendation available. * Significantly different from girls (*p* < 0.05). ** Significantly different from girls (*p* < 0.01). *** Significantly different from girls (*p* < 0.0001).

**Table 4:** Bone status of the study volunteers. Data are presented as Mean ± SD.

Bone Status Parameters	Girls (n = 248)	Boys (n = 231)
BUA (dB MHz^−1^)	111 ± 18	110 ± 16
BUA Z-score >−2.0 (%) ≤−2.0 (%)	3.55 ± 1.22 100 0	3.87 ± 1.12 100 0
SOS (m s^−1^)	1570 ± 28	1571 ± 34
SOS Z-score >−2.0 (%) ≤−2.0 (%)	−0.09 ± 0.98 98.0 2.0	0.37 ± 1.33 99.6 0.4
SI	94 ± 18	94 ± 19

BUA = broadband ultrasound attenuation, SOS = speed of sound, SI = stiffness index.

**Table 5:** Correlation coefficients (*r*) between bone parameters and several variables in girls (*n* = 248) and boys (*n* = 231).

Sex	BUA (dB MHz−1) *r* (*p*-Value)	SOS (m s−1) *r* (*p*-Value)	SI *r* (*p*-Value)
Age (years)			
Girls Boys	**0.543 (0.000)** **0.566 (0.000)**	**0.378 (0.000)** **0.398 (0.000)**	**0.523 (0.000)** **0.536 (0.000)**
BMI (kg m^−2^)			
Girls Boys	**0.477 (0.000)** **0.417 (0.000)**	**0.269 (0.000)** **0.165 (0.012)**	**0.435 (0.000)** **0.319 (0.000)**
Fat-free mass (kg)			
Girls Boys	**0.576 (0.000)** **0.618 (0.000)**	**0.360 (0.000)** **0.396 (0.000)**	**0.540 (0.000)** **0.563 (0.000)**
Fat mass (kg)			
Girls Boys	**0.451 (0.000)** **0.226 (0.001)**	**0.203 (0.001)** −0.053 (0.424)	**0.392 (0.000)** 0.094 (0.156)
Fat mass (%)			
Girls Boys	**0.325 (0.000)** −0.061 (0.359)	0.108 (0.091) **−0.266 (0.000)**	**0.270 (0.000)** **−0.182 (0.006)**
PAL ^a^			
Girls Boys	**0.171 (0.011)** **0.180 (0.017)**	**0.183 (0.006)** **0.240 (0.001)**	**0.196 (0.004)** **0.223 (0.003)**
Puberty category score ^b^			
Girls Boys	**0.548 (0.000)** **0.555 (0.000)**	**0.369 (0.000)** **0.452 (0.000)**	**0.532 (0.000)** **0.543 (0.000)**
BoneHEI			
Girls Boys	−0.061 (0.373) −0.109 (0.113)	−0.093 (0.169) −0.056 (0.421)	−0.075 (0.271) −0.083 (0.231)
Staying outside (h d^−1^)			
Girls Boys	0.052 (0.426) **0.136 (0.042)**	0.034 (0.607) 0.047 (0.485)	0.047 (0.468) 0.096 (0.150)
Duration of breastfeeding during infancy (months)			
Girls Boys	−0.068 (0.323) **0.172 (0.018)**	−0.090 (0.190) 0.098 (0.179)	−0.067 (0.327) **0.164 (0.023)**

Coefficients in bold indicate statistical significance. BoneHEI = Bone Healthy Eating Index, BMI = body mass index, BUA = broadband ultrasound attenuation, PAL = physical activity level, SOS = speed of sound, SI = stiffness index. ^a^ Data presented for 220 girls and 174 boys. ^b^ Data presented for 172 girls and 177 boys.

**Table 6:** Stepwise multiple regression analysis with stiffness index (SI) as dependent variable.

Regression Steps	*B*	*SE B*	*β*	*R^2^*
Step 1				0.28 ***
Constant	23.84	5.74		
Age (years)	5.21	0.42	0.53 ***	
Step 2				0.32 ***
Constant	28.43	5.68		
Age (years)	3.15	0.60	0.32 ***	
Fat-free mass	0.59	0.13	0.29 ***	
Step 3				0.33 ***
Constant	25.70	5.72		
Age (years)	2.88	0.60	0.29 ***	
Fat-free mass	0.69	0.13	0.34 ***	
Sex (0 = male, 1 = female)	4.31	1.57	0.12 **	
Step 4				0.35 ***
Constant	−0.60	10.95		
Age (years)	2.97	0.60	0.30 ***	
Fat-free mass	0.65	0.13	0.32 ***	
Sex (0 = male, 1 = female)	6.21	1.70	0.17 ***	
PAL	17.55	6.25	0.13 **	

PAL = physical activity level. ** *p* < 0.01. *** *p* < 0.0001.

## Supplementary Materials

The following is available online at https://www.mdpi.com/1660-4601/17/19/7294/s1, Questionnaire for school children.
