# Supplementary material for: Association between Body Composition, Physical Activity, Food Intake and Bone Status in German Children and Adolescents
Source: Int J Environ Res Public Health. 2020 Oct 6;17(19):7294. doi: 10.3390/ijerph17197294 (PMC7579391; doi:10.3390/ijerph17197294)
Supplement: Supplementary file 1 [file ijerph-17-07294-s001.pdf]

Questionnaire for school children

Project: Bone health and lifestyle of German school children

University of Education Schwäbisch Gmünd, Germany

Institute of Health Sciences

Prof. Dr. Petra Lührmann

Dr. Antje Schweter

Code: _ _ _ _ _ _ _

	**Example:**
1. First letter of the surname of your father	Surname father: **R**alf
2. First letter of the surname of your mother	Surname mother: **E**rika
3. Your month of birthday as two-digit number	Month of birthday: **03**
4. Number of siblings as two-digit number	Number of siblings: **01**
5. Last letter or your last name	Own last name: Schmid**t**
	**Code: R E 03 01 T**

All your information will be kept in confidence!

Personal data

1. **Sex**
   ☐Male ☐Female

2. **Date of birth**
   Month/Year ________ / _________

3. **Which school are you visiting?**
   ☐*Grundschule*
   ☐*Hauptschule*
   ☐*Realschule*
   ☐*Gymnasium*

4. **Which class are you joining?**
   __________ class

5. **In which country you were born?**
   ☐In Germany
   ☐In another country: ____________________

6. **Since when do you live in Germany?**
   ☐Since my birth
   ☐Since ___________________ (date)

7. **Are you smoking?**
   ☐Yes, I am smoking.
   ☐I am occasionally smoking.
   ☐I smoked, but stopped it.
   ☐No, I have never smoked.

Physical activity

8. **How long are you usually staying outside?**

   In summer:
   During the week: __________ hours per day
   During weekend days: __________ hours per day

   In winter:
   During the week: __________ hours per day
   During weekend days: __________ hours per day

9. **How many hours are you sleeping per day?**
   During the day: __________ hours During the night: __________ hours

10. **How much time do you spend for the following things per week?**

Activity	Hours per week
TV/video/DVD	___________
Video games	___________
Computer/internet	___________
Listening music	___________
Mobile phone	___________
Homework	___________

11. **How much time do you spend for the following activities per week?**

Activity	Hours per week
Housework and gardening	___________
Walking *(promenade, to school, shopping, etc.)*	___________
Riding a bike *(to school, shopping, etc.)*	___________
Outdoor playing	___________
Sports	
Light activities *(gymnastics, yoga, etc.)*	___________
Medium activities *(table tennis, inline skating, swimming, horse riding, tennis, dancing, etc.)*	___________
Exhaustive activities *(soccer, handball, volleyball, basketball, running)*	___________
Other activities, for example _____________________	___________

Questionnaire about your diet in the last few weeks

Please tick only one box per question.

12. **How often have you eaten/drunken in the last few weeks <u>milk</u>** (including chocolate milk, milk for cereals) **/<u>yoghurt</u>/<u>curd</u>/<u>buttermilk</u>?**

☐Never
☐once per month
☐2-3 times per month
☐1-2 times per week
☐3-4 times per week
☐5-6 times per week
☐once per day
☐2-3 times per day
☐4-5 times per day
☐more than 5 times per day

If yes, how much are you eating/drinking usually? (1 glass contains 200 mL)
☐¼ glass/cup or less
☐¼ glass/cup
☐1 glass/cup
☐2 glasses/cups
☐3 glasses/cups or more

13. How often have you eaten in the last few weeks <u>cheese</u> (soft cheese/ hard cheese)**?**

☐Never
☐once per month
☐2-3 times per month
☐1-2 times per week
☐3-4 times per week
☐5-6 times per week
☐once per day
☐2-3 times per day
☐4-5 times per day
☐more than 5 times per day

If yes, how much are you eating usually?
☐¼ slice/portion or less
☐½ slice/portion
☐1 slice/portion
☐2 slices/portion
☐3 slices/portions or more

14. How often have you eaten in the last few weeks <u>cream cheese?</u>

☐Never
☐once per month
☐2-3 times per month
☐1-2 times per week
☐3-4 times per week
☐5-6 times per week
☐once per day
☐2-3 times per day
☐4-5 times per day
☐more than 5 times per day

If yes, how much are you eating usually?
☐½ tablespoon or less
☐1 tablespoon (level)
☐2 tablespoons (level)
☐3 tablespoons (level)
☐4 tablespoons (level) or more

15. **How often have you drunken in the last few weeks <u>lemonade</u>** (coke/lemonade/soft drinks/energy drinks/iced tea)**?**

☐Never
☐once per month
☐2-3 times per month
☐1-2 times per week
☐3-4 times per week
☐5-6 times per week
☐once per day
☐2-3 times per day
☐4-5 times per day
☐more than 5 times per day

If yes, how much are you drinking usually? (1 glass contains 200 mL)
☐¼ glass or less
☐¼ glass
☐1 glass
☐2 glasses
☐3 glasses or more

16. **How often have you drunken in the last few weeks <u>coffee</u> / <u>black</u> / <u>green tea</u>?**

☐Never
☐once per month
☐2-3 times per month
☐1-2 times per week
☐3-4 times per week
☐5-6 times per week
☐once per day
☐2-3 times per day
☐4-5 times per day
☐more than 5 times per day

If yes, how much are you drinking usually? (1 cup contains 150 mL)
☐¼ cup or less
☐¼ cup
☐1 cup
☐2 cups
☐3 cups or more

17. **How often have you drunken in the last few weeks <u>fruit juice</u> / <u>fruit spritzer</u> / <u>fruit drinks</u>?**

☐Never
☐once per month
☐2-3 times per month
☐1-2 times per week
☐3-4 times per week

☐5-6 times per week
☐once per day
☐2-3 times per day
☐4-5 times per day
☐more than 5 times per day

If yes, how much are you drinking usually? (1 glass contains 200 mL)
☐¼ glass or less
☐¼ glass
☐1 glass
☐2 glasses
☐3 glasses or more

18. How often have you drunken in the last few weeks <u>mineral</u> / <u>tap water</u>?

☐Never
☐once per month
☐2-3 times per month
☐1-2 times per week
☐3-4 times per week
☐5-6 times per week
☐once per day
☐2-3 times per day
☐4-5 times per day
☐more than 5 times per day

If yes, how much are you drinking usually? (1 glass contains 200 mL)
☐¼ glass or less
☐¼ glass
☐1 glass
☐2 glasses
☐3 glasses or more

19. How often have you eaten in the last few weeks <u>bread</u> / <u>bread rolls</u>?

☐Never
☐once per month
☐2-3 times per month
☐1-2 times per week
☐3-4 times per week
☐5-6 times per week
☐once per day
☐2-3 times per day
☐4-5 times per day
☐more than 5 times per day

If yes, how much are you eating usually?
☐¼ slice/bread roll or less

☐¼ slice/bread roll
☐1 slice/bread roll
☐2 slices/bread rolls
☐3 slices/bread rolls or more

20. How often have you eaten in the last few weeks <u>eggs</u>?

☐Never
☐once per month
☐2-3 times per month
☐1-2 times per week
☐3-4 times per week
☐5-6 times per week
☐once per day
☐2-3 times per day
☐4-5 times per day
☐more than 5 times per day

If yes, how much are you eating usually?
☐¼ egg or less
☐¼ egg
☐1 egg
☐2 eggs
☐3 eggs or more

21. How often have you eaten in the last few weeks <u>meat</u> (without sausages)**?**

☐Never
☐once per month
☐2-3 times per month
☐1-2 times per week
☐3-4 times per week
☐5-6 times per week
☐once per day
☐2-3 times per day
☐4-5 times per day
☐more than 5 times per day

If yes, how much are you eating usually? (1 portion equals one handful)
☐1/8 portion/handful or less
☐¼ portion/handful
☐½ portion/handful
☐1 portion/handful
☐2 portions/handful or more

22. How often have you eaten in the last few weeks <u>sausages</u> / <u>ham</u>?

☐Never
☐once per month
☐2-3 times per month
☐1-2 times per week
☐3-4 times per week
☐5-6 times per week
☐once per day
☐2-3 times per day
☐4-5 times per day
☐more than 5 times per day

If yes, how much are you eating usually?
☐¼ slice or less
☐½ slice
☐1 slice
☐2 slices
☐3 slices or more

23. How often have you eaten in the last few weeks <u>fish</u>?

☐Never
☐once per month
☐2-3 times per month
☐1-2 times per week
☐3-4 times per week
☐5-6 times per week
☐once per day
☐2-3 times per day
☐4-5 times per day
☐more than 5 times per day

If yes, how much are you eating usually? (1 portion equals one handful)
☐1/3 portion/handful or less
☐½ portion/handful
☐1 portion/handful
☐2 portion/handful
☐3 portions/handful or more

24. How often have you eaten in the last few weeks <u>fruits</u> (fresh/boiled/preserved/frozen fruits)**?**

☐Never
☐once per month
☐2-3 times per month
☐1-2 times per week
☐3-4 times per week

☐5-6 times per week
☐once per day
☐2-3 times per day
☐4-5 times per day
☐more than 5 times per day

If yes, how much are you eating usually? (1 portion equals one handful)
☐¼ portion/handful or less
☐½ portion/handful
☐1 portion/handful
☐2 portion/handful
☐3 portions/handful or more

25. **How often have you eaten in the last few weeks <u>cooked vegetables</u>** (prepared from fresh/preserved/frozen vegetables) **/ <u>salad</u> / <u>raw vegetables</u>** (tomatoes, sweet pepper, cucumber, carrots)**?**

☐Never
☐once per month
☐2-3 times per month
☐1-2 times per week
☐3-4 times per week
☐5-6 times per week
☐once per day
☐2-3 times per day
☐4-5 times per day
☐more than 5 times per day

If yes, how much are you eating usually? (1 portion equals one handful)
☐¼ portion/handful or less
☐½ portion/handful
☐1 portion/handful
☐2 portion/handful
☐3 portions/handful or more

26. **How often have you eaten in the last few weeks <u>sweets</u>** (chocolate/chocolate bar/cake/pastry/cookies/drops/fruit gums)**?**

☐Never
☐once per month
☐2-3 times per month
☐1-2 times per week
☐3-4 times per week
☐5-6 times per week
☐once per day
☐2-3 times per day
☐4-5 times per day
☐more than 5 times per day

If yes, how much are you eating usually? (1 portion equals one handful)
☐¼ portion/handful or less
☐½ portion/handful
☐1 portion/handful
☐2 portion/handful
☐3 portions/handful or more

27. **How often have you eaten in the last few weeks <u>snacks</u>** (chips/salt sticks/cracker)**?**

☐Never
☐once per month
☐2-3 times per month
☐1-2 times per week
☐3-4 times per week
☐5-6 times per week
☐once per day
☐2-3 times per day
☐4-5 times per day
☐more than 5 times per day

If yes, how much are you eating usually? (1 portion equals one handful)
☐¼ portion/handful or less
☐½ portion/handful
☐1 portion/handful
☐2 portion/handful
☐3 portions/handful or more

Pubertal stage

The next questions are about changed that may happening to your body. These changes normally happen to different young people at different ages. If you do not understand a question or do not know the answer, just mark “I don´t know”.

28. **Would you say that your growth in height:**
☐Not yet started
☐Barely started
☐Definitely started
☐Seems complete
☐I don´t know

29. **And how about the growth of your body hair? (“Body hair” means hair at any place other than your head, such as under your arms.). Would you say that your body hair growth:**
☐Not yet started
☐Barely started
☐Definitely started
☐Seems complete
☐I don´t know

30. Have you noticed any skin changes, especially pimples?

☐Not yet started

☐Barely started

☐Definitely started

☐Seems complete

☐I don´t know

FORM FOR GIRLS:

31. Have you noticed that your breasts have begun to grow?

☐Not yet started

☐Barely started

☐Definitely started

☐Seems complete

☐I don´t know

32. Have you begun to menstruate (started to have your period)?

☐Yes ☐No

If yes, how old were you when you started to menstruate? ____________________ years

FORM FOR BOYS:

33. Have you noticed a deepening of your voice?

☐Not yet started

☐Barely started

☐Definitely started

☐Seems complete

☐I don´t know

34. Have you begun to grow hair on your face?

☐Not yet started

☐Barely started

☐Definitely started

☐Seems complete

☐I don´t know

Thank you very much!

Questionnaire for the parents of the school children

Project: Bone health and lifestyle of German school children

University of Education Schwäbisch Gmünd, Germany

Institute of Health Sciences

Prof. Dr. Petra Lührmann

Dr. Antje Schweter

All your information will be kept in confidence!

1. **In which country you were born?** (Please indicate for both parents!)

Mother:
☐In Germany
☐In another country: __________________

Father:
☐In Germany
☐In another country: _________________

2. **Since when are you living in Germany?** (Please indicate for both parents!)

Mother:
☐Since my birth
☐Since: ________ (date)

Father:
☐Since my birth
☐Since: ________ (date)

3. **Which graduation degree do you have?** (Please indicate for both parents!)

	**Mother**	**Father**
No school leaving degree	☐	☐
Hauptschulabschluss/Volksschulabschluss	☐	☐
Realschulabschluss	☐	☐
Abschluss Polytechnische Oberschule	☐	☐
Abitur	☐	☐
University degree	☐	☐
Others	______________________	_______________________

4. **Are you currently employed?** (Please indicate for both parents!)

	**Mother**	**Father**
Housewife/househusband	☐	☐
Unemployed	☐	☐
Part-time working	☐	☐
Full-time working	☐	☐

5. **Did you child receive vitamin D supplementation during the first year of life?**
☐No, never.
☐Yes, rarely.
☐Yes, often.
☐Yes, frequently.

6. **Does your child suffer from a disease?**
☐No ☐Yes

If yes, please specify kind of disease and since when the disease occurred.

____

____

7. **Did your child ever experience a bone fracture?**
☐No ☐Yes

If yes, please specify kind of fracture and the date when the fracture occurred.

____

____

8. **Does your child takes medication on a regular base?**
☐No ☐Yes

If yes, please specify kind of medication and reason for intake.

____

____

Thank you very much!
